# Representation of concussion subtypes in common postconcussion symptom-rating scales

**DOI:** 10.2217/cnc-2019-0005

**Published:** 2019-11-01

**Authors:** Angela Lumba-Brown, Jamshid Ghajar, Jordan Cornwell, O Josh Bloom, James Chesnutt, James R Clugston, Raina Kolluri, John J Leddy, Masaru Teramoto, Gerard Gioia

**Affiliations:** 1 Department of Emergency Medicine, Stanford University, Stanford, CA 94304, USA; 2 Department of Neurosurgery, Brain Performance Center, Stanford University, Stanford, CA 94304 USA; 3 Department of Emergency Medicine, Stanford University, Stanford, CA 94304, USA; 4 Carolina Sports Concussion Clinic, Cary, NC 27513, USA; 5 Departments of Family Medicine, Neurology, Oregon Health & Science University, Portland, OR & Rebound Orthopedics & Neurosurgery, Portland, OR 97239, USA; 6 Departments of Community Health & Family Medicine & Neurology, University of Florida, Gainesville, FL 32611, USA; 7 Department of Neurosurgery, Brain Performance Center, Stanford University, Stanford, CA 94304, USA; 8 UBMD Department of Orthopaedics & Sports Medicine, Jacobs School of Medicine & Biomedical Sciences, State University of New York at Buffalo, Buffalo, NY 14203, USA; 9 Division of Physical Medicine & Rehabilitation, University of Utah, Salt Lake City, UT 84108, USA; 10 Division of Pediatric Neuropsychology, Safe Concussion Outcome Recovery & Education Program, Children’s National Health System, Departments of Pediatrics & Psychiatry & Behavioral Sciences, George Washington University School of Medicine, Rockville, MD 20850, USA

**Keywords:** concussion, oculomotor, postconcussion symptoms, vestibular

## Abstract

**Aim:**

Postconcussion symptom-rating scales are frequently used concussion assessment tools that do not align directly with new expert, consensus-based concussion subtype classification systems. This may result in delays in concussion diagnosis, subspecialty referral and rehabilitative strategies.

**Objective:**

To determine the representation of subtype-directed symptomatology in common postconcussion symptom-rating scales.

**Methods:**

Literature review and expert consensus were used to compile commonly used concussion symptom-rating scales. Statistics were generated to describe the degree of representation of the consensus symptom set.

**Results:**

The percentage of symptoms representing each subtype/associated condition is low overall (15–26%). The ocular-motor (11%) and vestibular subtypes (19%) and cervical strain (5%)-associated condition were the most under-represented and also had the greatest unmet needs.

**Conclusion:**

Concussion subtypes do not have equal representation on commonly used concussion symptom-rating scales. There is a need for a subtype-directed symptom assessment to allow for increased accuracy of diagnosis and to guide management.

Over the last 20 years, multiple postconcussion symptom-rating scales have been developed to better classify and quantify the range of symptoms of mild traumatic brain injury (mTBI), inclusive of concussion [1]. These scales use symptom descriptors/items to measure the presence and severity of symptoms, typically via three–seven point dimensional scaling, allowing for additive, total item-calculated scores. The various scales have been constructed for different purposes and needs, resulting in variability in their items. Variations between commonly used postconcussion symptom-rating scales include preinjury assessment, specific descriptors, number of descriptors, severity assessed, self-reported versus parent or clinician-reported assessments, targeted age of respondents, tool validation and cost. In addition, some scales have been developed specifically to target the symptoms most evident in the early acute phase (e.g., within 72 h of injury) as opposed to the postacute and chronic phases of recovery.

Postconcussion scales are key clinical adjuncts in the diagnosis and prognosis of mTBI [2,3]. Higher symptom scale severity ratings have been correlated with a variety of functional outcomes including longer recovery times [4], vestibular/oculomotor/cognitive impairment [5], greater difficulties in returning to school [6] and neurophysiological outcomes such as blood oxygen-dependent signal changes in functional magnetic resonance imaging (fMRI) and alterations in cerebral blood flow [7,8].

Historically, psychometric analyses of various symptom scales identified four subdomains of concussion symptoms: physical, cognitive, emotional and sleep/fatigue [9–11]. Recent studies support that mTBIs are heterogeneous in clinical presentation across domains, resulting in thematic phenotypes that allow for subtype-targeted management [12–16]. A US Department of Defense-tasked expert workgroup classified postconcussive symptoms and defined nonmutually exclusive clinically relevant subtypes including: vestibular; ocular-motor; anxiety/mood; cognitive; and headache/migraine [15,17,18]. Additionally, two concussion-associated (exacerbating) conditions, sleep disturbance and cervical strain, have been recognized in conjunction with the five concussion subtypes and affect general recovery [15]. Sleep abnormalities are a common and direct effect of brain injury, influencing and adversely affecting other subtypes’ severity and recovery [19 15]. Recent pediatric concussion guidelines published by the CDC (GA, USA) recommend management and treatment of symptom categories aligning with the concussion subtypes, including headache, vestibulo-oculomotor, sleep, emotional and cognitive impairment [2]. The question arises as to how well the current clinical symptom rating scales contain the key symptoms or the full symptom set to adequately assist subtype classification [20]. The current study queries the representation of subtype-directed symptomatology in common postconcussion symptom-rating scales.

## Methodology

Eight commonly used clinical concussion symptom-rating scales were identified from literature review [21–23] and multidisciplinary, multi-institutional expert workgroup consensus [], including: The Rivermead Post Concussion Symptoms Questionnaire (RPCS) [24], Post-Concussion Symptom Scale (PCSS) [25], Graded Symptom Checklist (GSC) [26], Sports Concussion Assessment Tool – 5th Edition [27], Concussion Symptom Inventory (CSI) [28], Post-Concussion Symptom Inventory – Parent (PCSI–P) [29], Acute Concussion Evaluation [30] and the Health and Behavior Inventory (HBI) [31,32]. A recent systematic review of the current literature identified the RPCS and the PCSS as two of the most commonly reported symptoms scales for older adolescents and adults in the last 15 years [2,33].

Descriptive postconcussive symptoms were thematically categorized into the five clinical concussion subtypes and two associated conditions mentioned above for each of the eight common rating scales [34]. The items within the eight symptom-rating scales were examined relative to a larger set of symptom-items generated by an expert workgroup []. This expanded set of symptom items was generated to more fully capture the nature of the five subtypes and two associated conditions, based on the experience of the expert group. The final membership of the consensus symptom-item set, against which each of the rating scales was compared, was as follows: headache/migraine (11 symptoms), cognitive (16 symptoms), anxiety/mood (15 symptoms), ocular-motor (14 symptoms), vestibular (18 symptoms), sleep (9 symptoms) and cervical strain (8 symptoms).

Several statistics were generated to describe the degree of representation of the consensus symptom set. First, symptoms were examined by rating scale: the percentage of symptoms representing each subtype and associated condition was calculated for each rating scale. This statistic reflects how well each rating scale performed in capturing each of the subtype symptom sets. Second, item representation was examined by subtype and associated condition; the mean percentage of consensus symptoms represented by the rating scales collectively for each subtype and associated condition defines how well each subtype domain was defined across the rating scales. Finally, the total percent of items not represented by any of the symptom rating scales was calculated to reflect the unmet need.

## Results

Table 1 reports the symptom item representation across the five concussion subtypes and two associated conditions for the eight clinical postconcussion symptom-rating scales.

**Table 1.** Categorization of concussion symptom-rating scale descriptors by concussion subtypes and associated conditions.

Subtype (n) consensus symptoms	Rivermead	PCSS	Graded symptom checklist	SCAT5	CSI	PCSI–P	ACE	HBI (child)	Mean item representation	% items not represented	
**Headache/migraine (8)**											
– Headache	X	X	X	X	X	X	X	X		X	
– Light sensitivity	X	X	X	X	X	X	X			X	
– Noise sensitivity	X	X	X	X	X	X	X			X	
– Nausea						X	X	X		X	
– Vomiting						X	X			X	
– Head feeling heavy										0	
– Head pounding/pulsating										0	
– Pressure in head				X						X	
– Percent of consensus symptoms represented	38%	38%	38%	50%	38%	63%	63%	25%	44%	25%	13%
**Cognitive (14)**											
– Poor concentration	X	X		X	X	X	X	X		X	
– Easily distracted			X					X		X	
– Problems remembering		X	X	X	X	X	X	X		X	
– Feeling slowed down/taking longer to think	X	X	X	X	X	X	X			X	
– Taking longer to complete tasks										0	
– Feeling foggy		X	X	X	X	X	X			X	
– Difficulty with multitasking										0	
– Problems with organization										0	
– Not feeling ‘as sharp’										0	
– Confusion				X		X	X	X		X	
– Forgetfulness	X							X		X	
– Problems following directions								X		X	
– Hard to learn new things								X		X	
– Problems finishing things								X		X	
– Percent of consensus symptoms represented	21%	29%	29%	36%	29%	36%	36%	57%	34%	29%	11%
**Anxiety/mood (15)**											
– Sadness		X	X	X		X	X			X	
– Depressed mood/tearful	X									X	
– Irritability	X	X	X	X		X	X			X	
– More emotional(ly reactive)		X	X	X		X	X			X	
– Nervous/anxious		X	X	X		X	X			X	
– Fearful/scared										0	
– Personality changes			X							X	
– Agitated										0	
– Frustrated	X									X	
– Feeling panic										0	
– Stressed										0	
– Worried										0	
– Short tempered										0	
– Hopeless										0	
– Suicidal thoughts										0	
– Percent of consensus symptoms represented	20%	27%	33%	27%	0%	27%	27%	0%	20%	53%	13%
**Ocular-motor (13)**											
– Visual problems		X				X	X			X	
– Blurred vision	X		X	X	X	(X)		X		X	
– Double vision	X					(X)		X		X	
– Seeing stars			X							X	
– Eye strain										0	
– Difficulty focusing/looking at near objects										0	
– Difficulty reading, following lines along the page										0	
– Eyes feel tired/uncomfortable when reading or doing close work										0	
– Lose concentration when reading or doing close work										0	
– Double vision when reading or doing close work										0	
– See the words move, jump, swim or appear to float on the page when reading or doing close work										0	
– ‘Pulling or pressured feeling’ around eyes when reading or doing close work										0	
– Notice the words blurring or coming in and out of focus when reading or doing close work										0	
– Percent of consensus symptoms represented	15%	8%	15%	8%	8%	8%	8%	15%	11%	69%	4%
**Vestibular (17)**											
– Dizziness	X	X	X	X	X	X	X	X		X	
– Balance problems		X	X	X	X	X	X			X	
– Ringing in ears			X							X	
– Poor coordination			X							X	
– Loss of orientation										0	
– Nausea/vomiting	X	X	X	X	X	X	X			X	
– Clumsy						X				X	
– ‘Slow, wavy’-type dizziness										0	
– Room spinning								X		X	
– Feeling light headed								X		X	
– Uneasy/anxious/nervous in busy environment										0	
– Feel uneasy in crowds or busy settings										0	
– Feel unstable on feet										0	
– Have motion sickness										0	
– Looking up makes feel dizzy or makes dizziness worse										0	
– Bending over makes feel dizzy or makes dizziness worse										0	
– Quick movements of head make dizzy or make dizziness worse										0	
– Percent of consensus symptoms represented	12%	18%	29%	18%	18%	24%	18%	18%	19%	53%	5%
**Sleep (8)**											
– Sleep disturbance	X		X							X	
– Drowsiness		X	X	X	X	X	X			X	
– Sleeping more than usual		X	X			X	X			X	
– Sleeping less than usual		X				X	X			X	
– Trouble falling asleep		X		X		X	X			X	
– Fatigue	X	X	X	X	X	X	X	X		X	
– Wake up in the middle of the night or early in the morning										0	
– If lie down to rest in the afternoon will likely fall asleep										0	
– Percent of consensus symptoms represented	25%	63%	50%	38%	25%	63%	63%	13%	42%	25%	20%
**Cervical strain (7)**											
– Neck pain				X				X		X	
– Numbness/tingling							X			X	
– Neck stiffness										0	
– Cervicogenic headache										0	
– Neck tightness										0	
– Feel neck spasms										0	
– Difficulty reading due to neck pain										0	
– Percent of consensus symptoms represented	0%	0%	0%	14%	0%	0%	14%	14%	5%	71%	7%
– Total % representation	18%	24%	28%	24%	17%	28%	29%	21%	26%	45%	

ACE: Acute concussion evaluation; CSI: Concussion symptom inventory; HBI: Health and behavior inventory; PCSI–P: Post-concussion symptom inventory – parent; PCSS: Post-concussion symptom scale; SCAT5: Sport concussion assessment tool 5th edition.

### Rating scale

Examination by rating scale indicates that the percentage of symptom items representing each subtype/associated condition was low overall, varying between 15 and 26%. Variability exists, however, in the performance of the scales representing the different subtypes and associated conditions, ranging as low as 0% for cervical strain symptom items to as high as 56% for sleep symptom items. Most symptom scales include at least one symptom per subtype and associated condition, with the exception of two scales for the anxiety/mood subtype (CSI and HBI) and five scales for the cervical strain concussion-associated condition (RPCS, PCSS, GSC, CSI and PCSI–P).

### Subtype

Examination of symptom-item representation by subtype and associated condition indicated that the headache and sleep symptom items were most represented, although still under 50% across the eight scales (44 and 42%, respectively). The cognitive subtype items were the third most represented symptoms (34%). Cervical strain subtype was the most under-represented (5%) with only three rating scales reporting one symptom each. The ocular-motor (11%) and vestibular (19%) subtypes were also minimally represented on the existing rating scales, with most scales including three ocular-motor symptom items (visual problems, blurred vision and double vision) and three common vestibular items (dizziness, balance problems and nausea/vomiting). Four common symptom items were represented for the anxiety/mood subtype (sadness, irritability, more emotional and nervous/anxious), yet only 20% of the consensus items, on average, were represented. An overall mean item representation across the five subtypes and two associated conditions was 26%.

### Unmet needs

The subtypes were examined for the percentage of consensus symptom items that had no representation on any of the eight symptom scales. The cervical strain condition had the greatest unmet need (71% of consensus items not represented by any rating scale), followed by the ocular-motor subtype (69% items not included) and the vestibular (53%) and anxiety/mood subtypes (53%). The cognitive (29%), sleep (25%) and headache (25%) had the lowest unmet need among the eight scales.

## Discussion

This work examined eight commonly used clinical symptom scales with respect to their representation of a consensus list of symptoms for each subtype and associated condition. We found that the symptoms within the eight clinical symptom-rating scales represent only 26% of the consensus symptoms across the five concussion subtypes and two associated conditions. Variability exists in item membership across the symptom rating scales with significant gaps identified in most subtype domains. While the majority of rating scales contained at least one or more symptom descriptors for each subtype/associated condition, they frequently lacked a full set of descriptors informing a more complete subtype-targeted diagnosis. The eight scales differed in their overall representation of the subtype areas. The HBI and CSI did not include the emotional symptoms as they were developed for the acute stage of recovery, when arguably these symptoms are less overt. These two scales also had two of the lowest overall symptom representation, along with RPCS. The HBI rating scale includes, however, a higher representation of cognitive symptoms (57%) and a greater number of unique cognitive symptoms that the other scales did not have. The Acute Concussion Evaluation (29%), GSC (28%) and PCSI–P (28%) included the highest percentage of symptoms, with representation in all subtypes and sleep represented but, as with the other rating scales, lacked representation of cervical strain.

Examination of the subtypes and associated conditions *per se*, irrespective of the rating scale, revealed the highest symptom-item representation for the headache/migraine (44%) and sleep (42%) domains. The symptom domains of cervical strain (5%), ocular-motor (11%) and vestibular (19%) were significantly under represented. These latter domains are also the most recent to be recognized and studied as postinjury functional areas of concern, making their under representation within the clinical rating scales understandable. The symptom-item list for the anxiety/mood subtype was somewhat unique as it was expanded by the consensus group to include both acute and chronic symptom manifestation, resulting in only 26% of symptoms represented across the scales. This is particularly concerning since anxiety and mood issues are associated with prolonged recovery and risk of suicide is also increased postconcussion [35–37].

With respect to unmet needs, the consensus group identified a significant number of new symptom items for four subtype domains not present on any rating scale: anxiety/mood (53%), ocular-motor (69%), vestibular (53%) and cervical strain (71%).

These findings indicate the need to bolster our postconcussion symptom rating scales. While the majority of the scales contained at least one symptom for each subtype, many descriptors posited to be important by an expert workgroup were absent [38]. If clinicians are interested in assessing the seven domains appropriately, existing symptom rating scales may be supplemented with these additional items. Further, more general descriptors such as ‘fatigue’, could be understood in several ways, such as cognitive fatigue or sleep deprivation, and require further definition to be useful in targeting effective treatment.

Though previous studies on concussion recovery consisted predominantly of samples of male adolescent and young adult athletes who recovered in an average of 7–14 days [39], recent studies with a broader ranges of age and sex support a longer average recovery time frame up to 4 weeks post injury [40]. Longer recovery duration may also be attributed to a more comprehensive assessment for both diagnosing and recovery tracking, with the inclusion of concussion symptom subtypes, neurocognitive testing, vestibulo-oculomotor tracking [41] and recognition of the role of cervical strain. A more comprehensive, integrated concussion symptom/sign assessment tool kit that incorporates a full range of subtype symptomology may provide a more accurate and complete diagnosis, improve prognosis and guide treatment planning. Inadequate or incomplete diagnoses or under-representation of symptoms could cause clinicians to miss key relevant clinical factors that warrant treatment to avoid delayed recovery in patients with concussions.

## Conclusion

There is a need for a robust and cohesive concussion assessment that incorporates subtype signs and symptomology to provide accurate diagnosis and prognosis. Under-representation of key symptoms can lead to an inadequate diagnosis, and may adversely impact recovery in patients with concussion. Enhancing current clinical symptom scales with the proposed consensus symptom list would address this need and may improve concussion evaluation and treatment.

## Limitations & future perspective

This study was limited by the inability to individually analyze the vast number of existing concussion symptom-rating scales due to resource constraints. Eight commonly reported scales were studied, representing a potential selection effect. Expert consensus generated the target set of symptoms against which the rating scales were examined, which is subject to selection bias. Further, this study categorized symptom items by five concussion subtypes and two associated conditions; there may be other subtypes not represented here with clinical implications. We must also acknowledge that the purpose and development of some of the symptom scales influenced the inclusion of different symptoms, for example, the CSI included the most predictive symptoms at the early postinjury time point. Thus, the emotional and sleep symptoms were not included. The HBI was constructed similarly, whereas other measures (PCSS and PCSI) were constructed with the intent to track the full span of recovery. Future directions include the development and examination of concussion subtype-directed symptom assessments in the diagnosis and management of mTBI.

**Executive summary**

- Post-concussion symptom-rating scales are recommended for concussion diagnosis and assessment in children and adults.
- Recent concussion subtype classifications have variable representation in commonly used postconcussive symptom scales.
- There is a need for concussion subtype-directed symptom assessments to allow for increased accuracy of diagnosis and to guide management.
- Future directions include the development and examination of concussion subtype-directed symptom assessments in the diagnosis and management of mild traumatic brain injury.
